# Supplementary material for: SARS-CoV-2 can infect human embryos
Source: Sci Rep. 2022 Sep 14;12:15451. doi: 10.1038/s41598-022-18906-1 (PMC9472724; doi:10.1038/s41598-022-18906-1)
Supplement: Supplementary file 1 — Supplementary Figures. [file 41598_2022_18906_MOESM1_ESM.pdf]

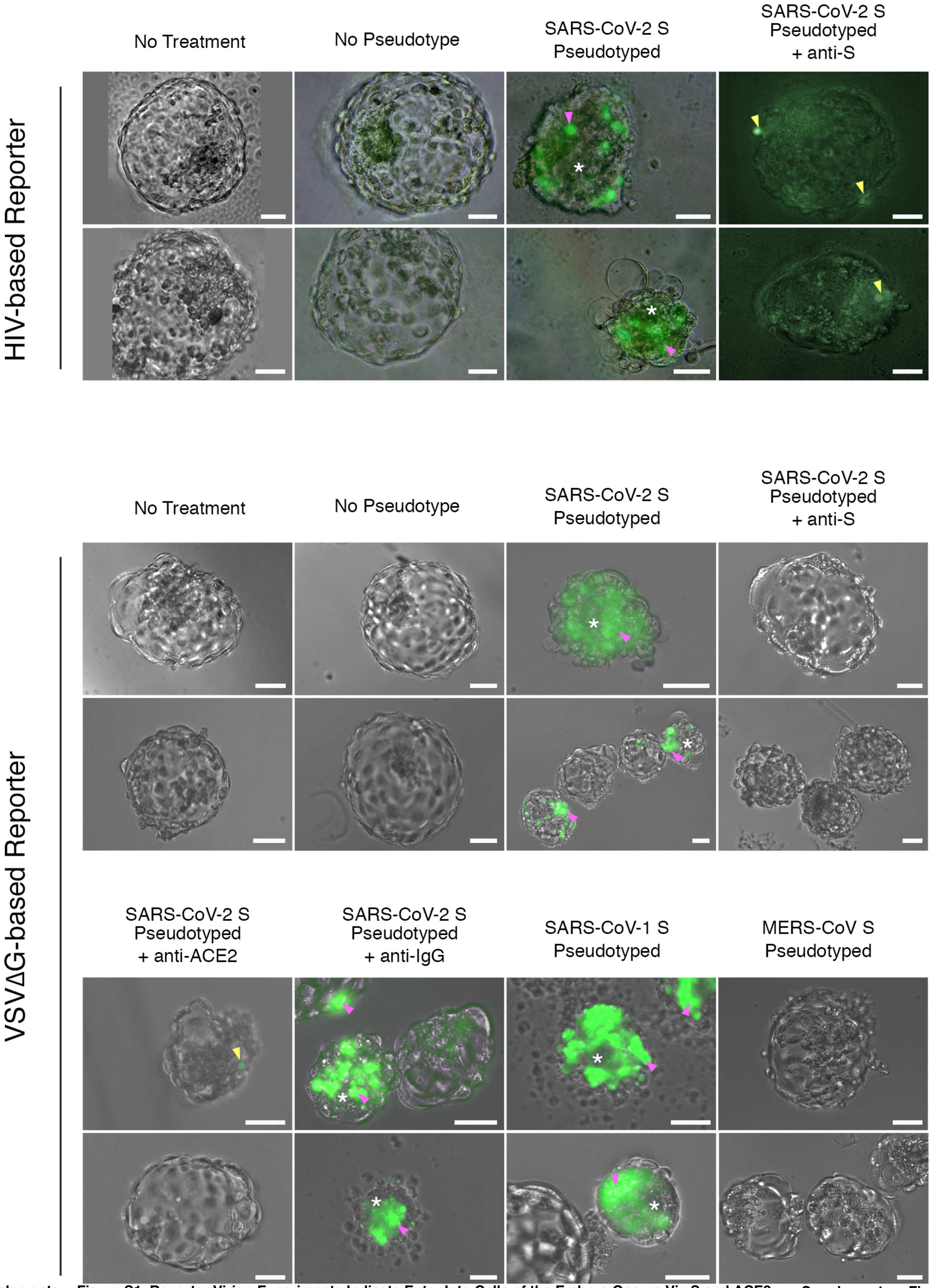

Supplementary Figure S1. Reporter Virion Experiments Indicate Entry Into Cells of the Embryo Occurs Via S and ACE2. **Supplementary Figure S1**

Sample images from GFP reporter virion experiments, displaying merged brightfield with epifluorescence signal. Top set shows results from HIV-based virus, bottom set shows results from the VSVΔG-based virus. Two representative images are shown per condition. The sample size of each condition is indicated in Table 1. Pink arrowheads point to cells displaying robust GFP signal, yellow arrowheads point to punctate GFP signal, and white asterisks indicate embryos manifesting poor health (likely due to expression of native genes in the reporter virions). Scale bars represent 50 μm.

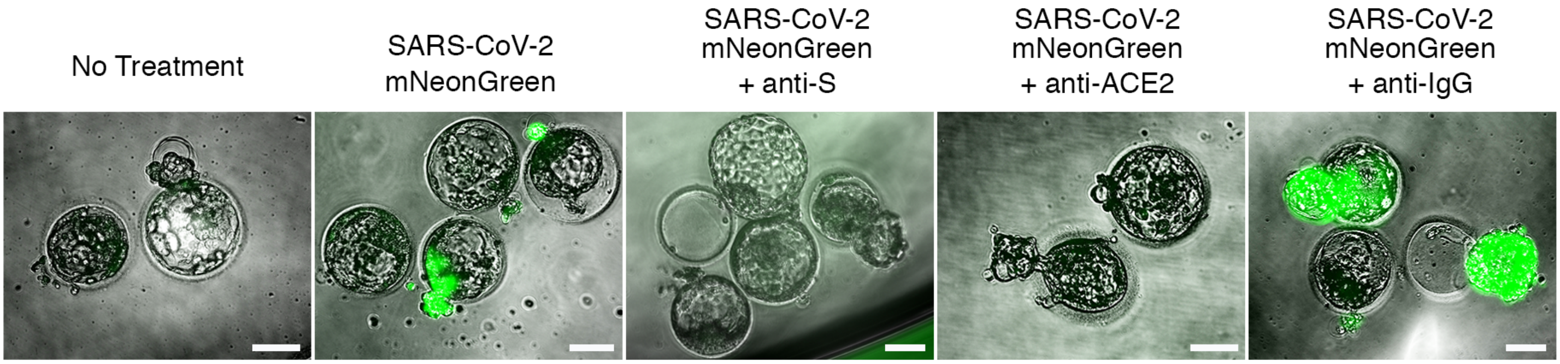

Supplementary Figure S2

Supplementary Figure S2. Live SARS-CoV-2 Experiments Indicate Susceptibility to Infection by Cells of the Embryo Through S and ACE2.
Sample images from SARS-CoV-2-mNeonGreen experiments, displaying merged brightfield with epifluorescence signal. The sample size of each condition is indicated in Table 2 and Supplementary Table S2. Scale bars represent 100 μm.
