# Supplementary material for: SARS-CoV-2 can infect human embryos
Source: Sci Rep. 2022 Sep 14;12:15451. doi: 10.1038/s41598-022-18906-1 (PMC9472724; doi:10.1038/s41598-022-18906-1)
Supplement: Supplementary file 2 — Supplementary Table S1. [file 41598_2022_18906_MOESM2_ESM.docx]

**Supplementary Table S1**

| Embryo Number | Ploidy | Affected Chromosomes | Ethnicity/Region |
| --- | --- | --- | --- |
| 1 | Euploid | - | Central American (Mexico) |
| 2 | Euploid | - | South Asian (India) |
| 3 | Euploid | - | African/Mediterranean |
| 4 | Euploid | - | European |
| 5 | Euploid | - | African/Mediterranean |
| 6 | Euploid | - | Middle Eastern (Jewish) |
| 7 | Euploid | - | European |
| 8 | Euploid | - | African/Mediterranean |
| 9 | Aneuploid | +21 | East Asian (China) |
| 10 | Aneuploid | +7,-21 | Central American/European |
| 11 | Aneuploid | -15 | Central American/European |
| 12 | Aneuploid | +5 | European |
| 13 | Aneuploid | -17 | South Asian (India) |
| 14 | Aneuploid | -21,-22 | Central American/European |
| 15 | Aneuploid | +7,+22 | East Asian (China) |
| 16 | Aneuploid | +4 | East Asian (Japan)/European |
| 17 | Aneuploid | +16,+20 | East Asian (China) |
| 18 | Aneuploid | -15,+21 | South-/East Asian (Vietnam/China) |
| 19 | Aneuploid | +6 | South East Asian (Indonesia)/European |
| 20 | Aneuploid | +15,+16 | East Asian (China) |
| 21 | Aneuploid | +11,+12,-22 | East Asian (China) |
| 22 | Aneuploid | -2 | East-/South Asian (China/India) |
| 23 | Aneuploid | +16 | South Asian (India) |
| 24 | Aneuploid | +15 | European |

**Supplementary Table S1. Features of Embryos used in RNA-seq Experiment.** Chromosomal status (euploid/aneuploid) as determined by PGT-A and background (ethnicity/region) of embryos tested.
