# Supplementary material for: SARS-CoV-2 can infect human embryos
Source: Sci Rep. 2022 Sep 14;12:15451. doi: 10.1038/s41598-022-18906-1 (PMC9472724; doi:10.1038/s41598-022-18906-1)
Supplement: Supplementary file 3 — Supplementary Table S2. [file 41598_2022_18906_MOESM3_ESM.docx]

**Supplementary Table S2**

SARS-CoV-2-mNeonGreen Virus

| Treatment | Number of Embryos Tested | Embryos w/ Fluorescent Cells |
| --- | --- | --- |
| No Treatment | 9 | 0 |
| No Treatment, No Hatching | 1 | 0 |
| Virus | 19 | 14 (73.7%) |
| Virus, No Hatching | 4 | 1 (25.0%) |
| Virus + anti-S neutralizing Antibody | 4 | 0 |
| Virus + anti-S neutralizing Antibody, No Hatching | 0 | 0 |
| Virus + anti-ACE2 neutralizing Antibody | 4 | 0 |
| Virus + anti-ACE2 neutralizing Antibody, No Hatching | 1 | 0 |
| Virus + anti-IgG neutralizing Antibody | 7 | 4 (57.1%) |
| Virus + anti-IgG neutralizing Antibody, No Hatching | 1 | 0 |

**Supplementary Table S2. Representation of Non-Hatching Embryos in Experimental Groups of the Live SARS-CoV-2 Experiments.** Breakdown of embryos by their (non-)hatching status.
